# Metformin and lung cancer risk in patients with type 2 diabetes mellitus

**DOI:** 10.18632/oncotarget.17066

**Published:** 2017-04-12

**Authors:** Chin-Hsiao Tseng

**Affiliations:** ^1^ Department of Internal Medicine, National Taiwan University College of Medicine, Taipei, Taiwan; ^2^ Division of Endocrinology and Metabolism, Department of Internal Medicine, National Taiwan University Hospital, Taipei, Taiwan; ^3^ Division of Environmental Health and Occupational Medicine of the National Health Research Institutes, Zhunan, Taiwan

**Keywords:** diabetes mellitus, lung cancer, metformin, Taiwan

## Abstract

This study evaluated whether metformin might reduce lung cancer risk. The reimbursement database of the Taiwan's National Health Insurance was used. A sample of 15414 never users and 280159 ever users of metformin (original sample) and a 1:1 matched-pairs of ever and never users (*n*=15414 in each group, matched sample) were recruited from patients with newly diagnosed type 2 diabetes mellitus during 1999-2005. They were followed until December 31, 2011. Cox regression incorporated with the inverse probability of treatment weighting using propensity score was used to estimate hazard ratios. Results showed that the respective incidence of lung cancer in ever and never users was 173.36 and 292.65 per 100000 person-years in the original sample; and was 211.71 and 292.65, respectively, in the matched sample. The overall hazard ratios (95% confidence intervals) of 0.586 (0.509-0.674) in the original sample and 0.717 (0.584-0.881) in the matched sample suggested a significantly lower risk among metformin users. Hazard ratios comparing the first (<22.60 months), second (22.60-46.67 months) and third (>46.67 months) tertile of cumulative duration of metformin use to never users was 1.163 (1.005-1.348), 0.612 (0.526-0.711) and 0.176 (0.148-0.210), respectively, in the original sample; and was 1.465 (1.131-1.897), 0.758 (0.566-1.016) and 0.228 (1.460-0.357) in the respective tertile of the matched sample. Sensitivity analyses after excluding patients with certain risk factors of cancer and subgroup analyses supported a favorable effect of metformin. In conclusion, metformin use may reduce lung cancer risk in patients with type 2 diabetes mellitus.

## INTRODUCTION

Diabetes mellitus significantly increases the risk of cancer and noncancer deaths [1–4]. According to global statistics, there were 1.8 million new cases of lung cancer in the world in 2012, accounting for about 13% of all cancer diagnoses [5]. In Taiwan, lung cancer represents approximately 15% and 8% of all cancers in men and women, respectively [6]. Although the incidence of lung cancer is decreasing in men and increasing in women in the whole world [5], it is increasing steadily in both sexes in Taiwan [6,7]. Smoking is the most important risk factor, but air pollution, fumes from cooking and other occupational and environmental exposure to carcinogens such as asbestos, arsenic, radon and polycyclic aromatic hydrocarbons are also potential risk factors [5].

Patients with type 2 diabetes mellitus (T2DM) suffer from a higher risk of various types of cancer including lung cancer [7,8]. Epidemiological studies show that metformin may reduce the risk of several types of cancer, including thyroid cancer [9], oral cancer [10], esophageal cancer [11], gastric cancer [12], colon cancer [13], kidney cancer [14], bladder cancer [15], prostate cancer [16], breast cancer [17], endometrial cancer [18], ovarian cancer [19] and cervical cancer [20]. However, whether metformin may reduce the risk of lung cancer remains to be answered.

There are some observational studies but the conclusions are inconsistent. Tsai et al. reported a reduced risk of lung cancer associated with metformin use [21], but Sakoda et al. did not find such a beneficial effect [22]. Meta-analyses also showed controversial results. In 2013, Wang et al. estimated a pooled relative risk of 0.91 (95% confidence interval: 0.80-1.03) from 7 observational studies [23]. In 2014, three meta-analyses concluded differently. Zhang et al. pooled the data from 4 studies and showed a significantly lower risk associated with metformin use (pooled relative risk: 0.71, 95% confidence interval: 0.55-0.95, *P*=0.02) [24]. However, by including 15 (11 cohort and 4 case-control) observational studies, Nie et al. reported a null association with an adjusted odds ratio of 0.99 (95% confidence interval: 0.87-1.12) [25]. The meta-analysis by Wu et al. (including 15 studies: 11 cohort, 2 case-control and 2 randomized controlled trials) suggested a 15% risk reduction (odds ratio 0.85, 95% confidence interval: 0.77-0.92), but this could not be demonstrated in smoking-adjusted subgroup [26]. In 2015, the latest meta-analysis including 8 observational studies by Zhu et al. suggested a significant 16% risk reduction (relative risk 0.84, 95% confidence interval: 0.73-0.97) [27].

By using the reimbursement databases of the Taiwan's National Health Insurance (NHI), the present study aimed at clarifying whether metformin use in patients with T2DM might reduce the risk of lung cancer. The tertiles of cumulative duration of metformin therapy were used to evaluate a dose-response relationship. To reduce “prevalent user bias” [28], only patients with newly diagnosed diabetes and new users of metformin were recruited. To reduce “immortal time bias” during the initial period of follow-up when the outcome can not occur [28], recruited patients should have been prescribed antidiabetic drugs for at least two times, and those who were followed up for <1 year were excluded. To reduce the residual confounding from the differences in baseline characteristics, Cox regression models incorporated with the inverse probability of treatment weighting (IPTW) using propensity score (PS) were created to estimate the hazard ratios [29] and additional analyses were conducted in a sample of 1:1 matched-pairs.

## RESULTS

**Figure 1:**
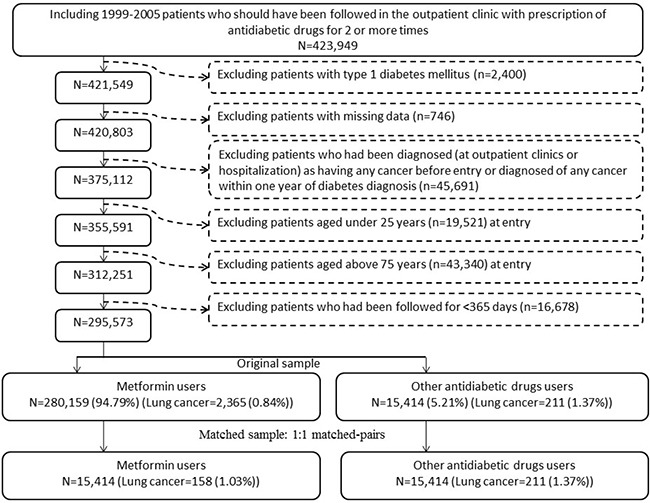
Flowchart showing the procedure in selecting the original sample and the matched sample into the study

There were 15414 never users and 280159 ever users of metformin in the original sample (Figure 1, Table 1). All characteristics differed significantly, except for hypertension, pioglitazone, Epstein-Barr virus (EBV)-related diagnoses and hepatitis B virus (HBV) infection (Table 1). There were 15414 never users and 15414 ever users in the matched-pairs (Figure 1, Table 1). Except for age, insulin, sulfonylurea, meglitinide and alcohol-related diagnoses, all other variables did not differ significantly between metformin ever and never users (Table 1). While examining the standardized differences, 11 of the variables had values >10% in the original sample, but none had a value >10% in the matched sample. Therefore, residual confounding from the variables was less likely in the matched sample.

**Table 1:** Characteristics of metformin never users and ever users

Variable	Original sample (N=295573)						Matched sample (N=30828)					
	Never users (n=15414)		Ever users (n=280159)		*P* value	SD	Never users(n=15414)		Ever users(n=15414)		*P* value	SD
	n	%	n	%			n	%	n	%		
**Demographic data**												
Age (years)*	63.76	10.41	61.43	10.21	<0.0001	-23.07	63.76	10.41	64.11	9.87	0.0020	4.23
Sex (men)	8819	57.21	150517	53.73	<0.0001	-7.27	8819	57.21	8767	56.88	0.5496	-0.92
Occupation												
I	6033	39.14	113083	40.36	<0.0001		6033	39.14	5952	38.61	0.5105	
II	3075	19.95	64289	22.95		7.66	3075	19.95	3074	19.94		-0.05
III	3236	20.99	54670	19.51		-3.68	3236	20.99	3341	21.68		1.85
IV	3070	19.92	48117	17.17		-7.48	3070	19.92	3047	19.77		-0.49
Living region												
Taipei	5210	33.80	94703	33.80	<0.0001		5210	33.80	5198	33.72	0.6683	
Northern	1565	10.15	33569	11.98		6.01	1565	10.15	1570	10.19		-0.03
Central	2681	17.39	49963	17.83		1.23	2681	17.39	2615	16.97		-1.14
Southern	2692	17.46	44872	16.02		-4.01	2692	17.46	2782	18.05		1.68
Kao-Ping and Eastern	3266	21.19	57052	20.36		-2.01	3266	21.19	3249	21.08		0.00
**Major comorbidities**												
Hypertension	12627	81.92	228657	81.62	0.3456	-0.81	12627	81.92	12650	82.07	0.7332	0.60
Dyslipidemia	11209	72.72	233135	83.22	<0.0001	26.93	11209	72.72	11139	72.27	0.3720	-0.61
Obesity	413	2.68	16082	5.74	<0.0001	15.36	413	2.68	376	2.44	0.1821	-1.36
**Diabetes-related complications**												
Nephropathy	5330	34.58	75028	26.78	<0.0001	-18.17	5330	34.58	5335	34.61	0.9523	-0.64
Eye disease	2911	18.89	87493	31.23	<0.0001	29.15	2911	18.89	2786	18.07	0.0666	-2.54
Stroke	5085	32.99	80963	28.90	<0.0001	-9.35	5085	32.99	5180	33.61	0.2509	1.27
Ischemic heart disease	7352	47.70	127487	45.51	<0.0001	-4.58	7352	47.70	7324	47.52	0.7495	-0.23
Peripheral arterial disease	3604	23.38	70404	25.13	<0.0001	4.23	3604	23.38	3583	23.25	0.7773	-0.43
**Antidiabetic drugs**												
Insulin	1243	8.06	5814	2.08	<0.0001	-29.03	1243	8.06	943	6.12	<0.0001	-9.02
Sulfonylurea	11298	73.30	185722	66.29	<0.0001	-12.17	11298	73.30	11844	76.84	<0.0001	9.58
Meglitinide	1248	8.10	10100	3.61	<0.0001	-20.17	1248	8.10	1111	7.21	0.0033	-3.14
Acarbose	1713	11.11	14193	5.07	<0.0001	-21.80	1713	11.11	1654	10.73	0.2813	-2.19
Rosiglitazone	469	3.04	12833	4.58	<0.0001	8.46	469	3.04	441	2.86	0.3461	-1.29
Pioglitazone	391	2.54	6779	2.42	0.3582	-0.09	391	2.54	389	2.52	0.9422	-0.39
**Potential risk factors of cancer**												
COPD	7659	49.69	136662	48.78	0.0280	-2.17	7659	49.69	7640	49.57	0.8287	-0.18
Tobacco abuse	431	2.80	11028	3.94	<0.0001	6.47	431	2.80	403	2.61	0.3256	-1.13
Alcohol-related diagnoses	1178	7.64	19452	6.94	0.0009	-3.36	1178	7.64	1083	7.03	0.0379	-2.79
History of HP infection	5141	33.35	84180	30.05	<0.0001	-7.84	5141	33.35	5102	33.10	0.6372	-0.92
EBV-related diagnoses	109	0.71	1997	0.71	0.9352	0.06	109	0.71	105	0.68	0.7838	-0.31
HBV infection	681	4.42	11704	4.18	0.1469	-1.53	681	4.42	631	4.09	0.1583	-1.84
HCV infection	998	6.47	14304	5.11	<0.0001	-6.39	998	6.47	951	6.17	0.2714	-1.35
**Medications that are commonly used in diabetes patients and may affect cancer risk**												
ACEI/ARB	10731	69.62	204108	72.85	<0.0001	7.37	10731	69.62	10657	69.14	0.3605	-0.95
Calcium channel blocker	9705	62.96	166164	59.31	<0.0001	-7.70	9705	62.96	9770	63.38	0.4428	0.96
Statin	8373	54.32	184811	65.97	<0.0001	24.99	8373	54.32	8299	53.84	0.3977	-0.88
Fibrate	5303	34.40	120199	42.90	<0.0001	18.18	5303	34.40	5259	34.12	0.5975	-0.36
Aspirin	8849	57.41	171328	61.15	<0.0001	7.81	8849	57.41	8839	57.34	0.9083	0.04

*Age is expressed as mean and standard deviation

Refer to Materials and Methods for the classification of occupation

SD: standardized difference, COPD: chronic obstructive pulmonary disease, HP: *Helicobacter pylori*, EBV: Epstein-Barr virus, HBV: hepatitis B virus, HCV: hepatitis C virus, ACEI/ARB: angiotensin converting enzyme inhibitor/angiotensin receptor blocker

Table 2 shows the incidences of lung cancer by metformin exposure and the hazard ratios comparing exposed to unexposed patients in the original sample and the matched sample, respectively. The respective incidence of lung cancer for ever users and never users was 173.36 and 292.65 per 100,000 person-years in the original sample; and was 211.71 and 292.65 per 100,000 person-years in the matched sample. The overall hazard ratio (95% confidence interval) of 0.586 (0.509-0.674) in the original sample and 0.717 (0.584-0.881) in the matched sample suggested a significantly lower risk of lung cancer associated with metformin use. When examining lung cancer by the tertiles of cumulative duration, there was a trend of decreasing incidence with longer duration of exposure. A significantly lower risk was observed for the third tertiles, but the first tertiles were associated with a significantly higher risk, in both the original sample and the matched sample.

**Table 2:** Incidences of lung cancer and hazard ratios by metformin exposure

Metformin use	*n*	*N*	Person-years	Incidence rate (per 100,000 person-years)	HR	95% CI	*P* value
**Original sample**							
Never users	211	15414	72099.19	292.65	1.000		
Ever users	2365	280159	1364192.16	173.36	0.586	(0.509-0.674)	<0.0001
**Tertiles of cumulative duration of metformin therapy (months)**							
Never users	211	15414	72099.19	292.65	1.000		
<22.60	1190	92347	358747.29	331.71	1.163	(1.005-1.348)	0.0433
22.60-46.67	854	92759	464388.82	183.90	0.612	(0.526-0.711)	<0.0001
≥46.67	321	95053	541056.05	59.33	0.176	(0.148-0.210)	<0.0001
**Matched sample**							
Never users	211	15414	72099.19	292.65	1.000		
Ever users	158	15414	74631.29	211.71	0.717	(0.584-0.881)	0.0016
**Tertiles of cumulative duration of metformin therapy (months)**							
Never users	211	15414	72099.19	292.65	1.000		
<22.60	80	5085	19517.80	409.88	1.465	(1.131-1.897)	0.0038
22.60-47.13	57	5088	25246.49	225.77	0.758	(0.566-1.016)	0.0641
≥47.13	21	5241	29867.01	70.31	0.228	(1.460-0.357)	<0.0001

*n*: case number of incident lung cancer, *N*: case number followed

HR: hazard ratio, CI: confidence interval

Table 3 shows the overall hazard ratios in the original sample as sensitivity analyses after excluding patients with certain risk factors of cancer. In all analyses, a significantly lower risk of lung cancer associated with metformin use was observed.

**Table 3:** Sensitivity analyses estimating hazard ratios for lung cancer for ever versus never users of metformin after excluding patients with certain risk factors of cancer

Model	*n*/*N* in ever users	*n*/*N* in never users	HR	95% CI	*P* value
Excluding patients who developed other cancers during follow-up	2365 / 262556	211 / 14150	0.578	(0.502-0.665)	<0.0001
Excluding patients with COPD/tobacco abuse	751 / 138825	61 / 7583	0.649	(0.500-0.842)	0.0012
Excluding patients with alcohol-related diagnoses	2165 / 260707	198 / 14236	0.570	(0.493-0.660)	<0.0001
Excluding patients with HP infection	1546 / 195979	135 / 10273	0.578	(0.484-0.689)	<0.0001
Excluding patients with EBV-related diagnoses	2346 / 278162	207 / 15305	0.593	(0.514-0.683)	<0.0001
Excluding patients with HBV/HCV infection	2152 / 255930	198 / 13864	0.561	(0.485-0.649)	<0.0001

*n*: case number of incident lung cancer, *N*: case number followed

HR: hazard ratio, CI: confidence interval

COPD: chronic obstructive pulmonary disease, HP: Helicobacter pylori, EBV: Epstein-Barr virus, HBV: hepatitis B virus, HCV: hepatitis C virus

Table 4 shows the hazard ratios in different subgroups of age, sex, follow-up duration, the presence or absence of diagnoses of chronic obstructive pulmonary disease (COPD)/tobacco abuse, and the use of insulin, sulfonylurea, meglitinide, acarbose, rosiglitazone, pioglitazone, angio-tensin converting enzyme inhibitor/angiotensin receptor blocker (ACEI/ARB), calcium channel blocker, statin, fibrate and aspirin. All hazard ratios favored a lower risk of lung cancer associated with metformin use, even though the *P*-values were not significant for the subgroups who had been followed up for ≥5 years or had been using insulin, acarbose or rosiglitazone.

**Table 4:** Subgroup analyses estimating hazard ratios for lung cancer for ever versus never users of metformin

Model	*n*/*N* in ever users	*n*/*N* in never users	HR	95% CI	*P* value
Age <65 years	1001 / 174601	105 / 7958	0.503	(0.403-0.628)	<0.0001
Age ≥65 years	1364 / 105558	126 / 7456	0.734	(0.611-0.880)	0.0009
Men	1541 / 150517	133 / 8819	0.649	(0.544-0.775)	<0.0001
Women	824 / 129642	78 / 6595	0.507	(0.402-0.639)	<0.0001
Follow-up duration <5 years	2014 / 102702	184 / 6200	0.557	(0.479-0.647)	<0.0001
Follow-up duration ≥5 years	351 / 177457	27 / 9214	0.694	(0.469-1.027)	0.0676
COPD and/or tobacco abuse: (+)	1614 / 141334	150 / 7831	0.557	(0.472-0.659)	<0.0001
COPD and/or tobacco abuse: (-)	751 / 138825	61 / 7583	0.649	(0.500-0.842)	0.0012
Insulin use (+)	42 / 5814	13 / 1243	0.629	(0.337-1.171)	0.1437
Insulin use (-)	2323 / 274345	198 / 14171	0.581	(0.502-0.672)	<0.0001
Sulfonylurea use (+)	1641 / 185722	162 / 11298	0.581	(0.494-0.682)	<0.0001
Sulfonylurea use (-)	724 / 94437	49 / 4116	0.606	(0.454-0.810)	0.0007
Meglitinide use (+)	96 / 10100	19 / 1248	0.538	(0.329-0.879)	0.0135
Meglitinide use (-)	2269 / 270059	192 / 14166	0.593	(0.512-0.687)	<0.0001
Acarbose use (+)	147 / 14193	18 / 1713	0.857	(0.525-1.398)	0.5368
Acarbose use (-)	2218 / 265966	193 / 13701	0.566	(0.489-0.656)	<0.0001
Rosiglitazone use (+)	117 / 12833	7 / 469	0.587	(0.274-1.258)	0.1709
Rosiglitazone use (-)	2248 / 267326	204 / 14945	0.586	(0.508-0.677)	<0.0001
Pioglitazone use (+)	41 / 6779	7 / 391	0.283	(0.127-0.630)	0.0020
Pioglitazone use (-)	2324 / 273380	204 / 15023	0.597	(0.517-0.688)	<0.0001
ACEI/ARB use (+)	1784 / 204108	155 / 10731	0.568	(0.482-0.669)	<0.0001
ACEI/ARB use (-)	581 / 76051	56 / 4683	0.626	(0.476-0.824)	0.0008
Calcium channel blocker use (+)	1557 / 166164	144 / 9705	0.592	(0.499-0.702	<0.0001
Calcium channel blocker use (-)	808 / 113995	67 / 5709	0.585	(0.456-0.751)	<0.0001
Statin use (+)	1392 / 184811	85 / 8373	0.706	(0.567-0.879)	0.0018
Statin use (-)	973 / 95348	126 / 7041	0.549	(0.456-0.661)	<0.0001
Fibrate use (+)	949 / 120199	67 / 5303	0.579	(0.452-0.742)	<0.0001
Fibrate use (-)	1416 / 159960	144 / 10111	0.601	(0.506-0.714)	<0.0001
Aspirin use (+)	1530 / 171328	119 / 8849	0.619	(0.514-0.746)	<0.0001
Aspirin use (-)	835 / 108831	92 / 6565	0.534	(0.430-0.662)	<0.0001

*n*: case number of incident lung cancer, *N*: case number followed

HR: hazard ratio, CI: confidence interval

COPD: chronic obstructive pulmonary disease, ACEI/ARB: angiotensin converting enzyme inhibitor/angiotensin receptor blocker

## DISCUSSION

The findings supported a significantly lower risk of lung cancer in patients with T2DM who used metformin (Tables 2-4).

The mechanisms for a reduced risk of lung cancer associated with metformin use remain to be explored. In general, metformin may exert its anticancer effect through the inhibition of tumor angiogenesis [30], suppressing cancer cell metabolism [31], activation of apoptosis and autophagy [32], inhibition of mammalian target of rapamycin (mTOR) [33], immunomodulation by increasing the number of CD8^+^ tumor-infiltrating lymphocytes [34], and impairing one-carbon metabolism acting like an antifolate drug [35].

Some *in vitro* and *in vivo* studies conducted in lung cancer cells specifically supported these potential mechanisms. The mTOR pathway is upregulated in non-small-cell lung cancer (NSCLC) and metformin inhibits its signaling by directly activating 5' adenosine monophosphate-activated protein kinase (AMPK) via liver kinase B1 (LKB1) [36]. Metformin may also inhibit the growth of human NSCLC cells by activating AMPK via an LKB1-independent pathway [37]. In *in vitro* studies, metformin blocks the M2-like polarization of macrophages (important for cancer progression and metastasis) and inhibits metastasis of Lewis lung cancer [38]. Metformin can also act in combination with salinomycin (a putative stem cell killer) to eradicate the NSCLC monolayer cells [39] and sensitize lung cancer cells to chemotherapeutic agents [40]. A meta-analysis suggested that metformin therapy is associated with an improved outcome in lung cancer patients with diabetes [41].

There is a higher risk of lung cancer in patients with obesity [42]. It is interesting to observe an increased risk in the first tertiles of cumulative duration of metformin therapy (Table 2). A residual confounding from obesity possibly explained such a result because metformin is considered as the first-line treatment for patients with T2DM, especially in those with obesity. Patients categorized in the first tertiles were short-term users and would be characterized by obesity when metformin was preferentially used. The increased risk associated with obesity in patients who were previously on diet control or treated with other medications might be carried over to these short-term users.

The present study has several strengths. First, all claims records of outpatient visits and hospital admission were included and the diagnoses were considered from both sources. Second, most medical co-payments can be waived by the NHI in patients with cancer, and there is a low drug cost-sharing in patients with certain conditions, such as those with a low-income household, veterans or patients with prescription refills for chronic disease. Therefore, the detection rate of lung cancer would be less biased by different social classes. Third, self-reporting bias was much reduced by the use of medical records.

The study limitations included a lack of actual measurement data for confounders such as anthropometric factors, smoking, alcohol drinking, family history, lifestyle, nutritional status, dietary pattern, and genetic parameters. In addition, we did not have exposure data of some occupational and environmental carcinogens and could not evaluate the impact of biochemical data. Another limitation is the lack of information on the pathology, grading and staging of lung cancer. Because adenocarcinoma represents 42% and 71% of all cases of lung cancer in men and women, respectively, in Taiwan [43], the findings should better be applied to adenocarcinoma rather than to squamous cell carcinoma, especially in females.

In summary, this study supports that metformin use in Taiwanese patients with T2DM may significantly reduce the risk of lung cancer, especially when it has been used for more than 4 years.

## MATERIALS AND METHODS

The NHI implemented in Taiwan since March 1995 is a compulsory and universal system of health insurance. It covers >99% of Taiwan residents and has contracts with >98% of the hospitals nationwide. The reimbursement databases are handled by the National Health Research Institutes and can be used for academic researches after proposal review and approval by an ethic review board. This study was granted with an approval number 99274.

Individuals were de-identified for the protection of privacy. Diabetes was coded 250.XX and lung cancer 162, based on the *International Classification of Diseases, Ninth Revision, Clinical Modification* (ICD-9-CM).

Figure 1 shows the procedures in selecting a cohort of patients with newly diagnosed T2DM (original sample) and a 1:1 matched-pairs of sample (matched sample) into the study. The patients should have been diagnosed as having diabetes at an onset age of 25-74 years during the period from 1999 to 2005. Patients with diabetes mellitus diagnosed during 1996-1998 were excluded to assure a first diagnosis of diabetes after 1999, and they should have been followed up in the outpatient clinic with prescription of antidiabetic drugs for 2 or more times (n=423949). In Taiwan, patients with type 1 diabetes can be waived of much of the co-payment after a certified diagnosis with issuance of a so-called “Severe Morbidity Card”. These patients with type 1 diabetes (n=2400) were excluded because metformin is not indicated for them. Patients with missing data (n=746), with a diagnosis of any cancer before entry or within one year of diabetes diagnosis (n=45691), aged <25 (n=19521) or ≥75 (n=43340) years, and followed up for <365 days (n=16678) were also excluded. As a result, there were 280159 patients who had ever been treated with metformin and 15414 patients who had never been treated with metformin (the original sample).

Because the characteristics might be imbalanced between metformin ever users and never users in the original sample, additional analyses were conducted in a sample of 1:1 PS matched-pairs (the matched sample). The matched sample was created by using the Greedy 8 → 1 digit match algorithm proposed by Parsons (the matching macro using SAS statistical software is available online) [44]. The PS was derived from all characteristics listed in Table 1 plus the date of entry by using logistic regression. Because the case number of never users was much smaller than ever users in the original sample, the number of matched-pairs of ever and never users was based on the case number of never users in the original sample (i.e., n=15414). Therefore, one case in the pool of ever users in the original sample was selected as a matched-pair to each of the never users. According to the matching algorithm, the best match with the highest 8 digits of the PS was first selected. If a matched-pair was made, no more matching was considered. If not, ever users would then be matched on 7 digits of the PS to the never users. The procedures were repeated sequentially to the lowest digit of PS until a matched-pair was made [44]. This matching method has been used in our previous studies [10, 12, 20, 45, 46].

Cumulative duration (months) of metformin use was calculated and its tertiles were used. Demographic data of age, sex, occupation and living region, and factors that might be correlated with metformin use, diabetes severity or cancer risk were considered as potential confounders. The living region and occupation were classified as detailed elsewhere [13]. In brief, the living region was classified as Taipei, Northern, Central, Southern, and Kao-Ping/Eastern. Occupation was classified as class I (civil servants, teachers, employees of governmental or private businesses, professionals and technicians), class II (people without a specific employer, self-employed people or seamen), class III (farmers or fishermen) and class IV (low-income families supported by social welfare, or veterans).

Other confounders included 1) major comorbidities associated with diabetes mellitus: hypertension (ICD-9-CM code: 401-405), dyslipidemia (272.0-272.4) and obesity (278); 2) diabetes-related complications: nephropathy (580-589), eye disease (250.5, 362.0, 369, 366.41 and 365.44), stroke (430-438), ischemic heart disease (410-414), and peripheral arterial disease (250.7, 785.4, 443.81 and 440-448); 3) antidiabetic drugs: insulin, sulfonylurea, meglitinide, acarbose, rosiglitazone and pioglitazone; 4) potential risk factors of cancer: COPD (a surrogate of smoking; 490-496), tobacco abuse (305.1, 649.0 and 989.84), alcohol-related diagnoses (291, 303, 535.3, 571.0-571.3 and 980.0), history of *Helicobacter pylori* (HP) infection (defined below), diagnoses related to EBV infection (075, 710.3 and 710.4), HBV infection (070.22, 070.23, 070.32, 070.33 and V02.61) and hepatitis C virus infection (070.41, 070.44, 070.51, 070.54 and V02.62); and 5) medications that are commonly used in diabetes patients and may potentially affect cancer risk: ACEI/ARB, calcium channel blocker, statin, fibrate and aspirin. History of HP infection was defined based on one of the following two criteria: 1) having received an HP eradication therapy (detailed previously [47] and defined in brief as a combination use of proton pump inhibitors or H2 receptor blockers, plus clarithromycin, metronidazole or levofloxacin, plus amoxicillin or tetracycline, with or without bismuth, in the same prescription order for 7-14 days); and/or 2) HP infection diagnosis (041.86).

The characteristics between never users and ever users were compared by Student's t test for age and by Chi-square test for other variables. The standardized differences proposed by Austin and Stuart as a test for balance diagnostics were calculated for all covariates [48]. A value of >10% might indicate potential confounding from the variable [48].

The incidence density of lung cancer was calculated for never users, ever users and tertiles of cumulative duration of metformin therapy. The numerator was the case number of incident lung cancer during follow-up, and the denominator was the person-years of follow-up. Follow-up started on the first day of the use of antidiabetic drugs and ended on December 31, 2011, at the time of a new diagnosis of lung cancer, or on the date of death or the last reimbursement record.

The treatment effect was estimated by Cox regression incorporated with IPTW using the PS [29]. Hazard ratios were estimated for ever versus never users and for each tertile of cumulative duration of metformin therapy using never users as referent.

Sensitivity analyses were conducted in the original sample by estimating the overall hazard ratios for ever versus never users after excluding patients with certain risk factors of cancer. These analyses were conducted after excluding 1) patients who developed other cancers during follow-up; 2) patients with COPD/tobacco abuse; 3) patients with alcohol-related diagnoses; and 4) patients with infections of HP, EBV and HBV/HCV, respectively.

Subgroup analyses were also conducted to examine the consistency of the findings. Hazard ratios were estimated for subgroups of age <65 years, age ≥65 years, men, women, follow-up duration < 5 years, follow-up duration ≥5 years, COPD/tobacco abuse (+), COPD/tobacco abuse (-) and with and without the use of certain medications (i.e., insulin, sulfonylurea, meglitinide, acarbose, rosiglitazone, pioglitazone, ACEI/ARB, calcium channel blocker, statin, fibrate and aspirin).

Analyses were conducted using SAS statistical software, version 9.3 (SAS Institute, Cary, NC). *P* < 0.05 was considered statistically significant.
